# Test System for Studying Biotin Transport upon SLC5A6 Gene Inactivation

**DOI:** 10.32607/actanaturae.27645

**Published:** 2025

**Authors:** A. Yu. Rudenko, P. A. Zotova, O. A. Averina, A. V. Priymak, M. P. Rubtsova, S. S. Mariasina, R. M. Ozhiganov, O. A. Dontsova, P. V. Sergiev

**Affiliations:** Belozersky Institute of Physico-Chemical Biology, Lomonosov Moscow State University, Moscow, 119192 Russia; Faculty of Fundamental Medicine, Lomonosov Moscow State University, Moscow, 119192 Russia; Department of Chemistry, Lomonosov Moscow State University, Moscow, 119192 Russia; Shemyakin-Ovchinnikov Institute of Bioorganic Chemistry, Russian Academy of Sciences, Moscow, 117997 Russia; Research and Educational Resource Center “Pharmacy”, RUDN University, Moscow, 117198 Russia; Higher Chemical College RAS, Mendeleev University of Chemical Technology, Moscow, 125190 Russia; Center for Molecular and Cellular Biology, Skolkovo Institute of Science and Technology, Moscow, 121205 Russia

**Keywords:** biotin, SLC5A6, hSMVT, biotin transport, cell membrane, test system, biotin derivatives

## Abstract

This paper introduces a test system for the investigation of biotin transport following inactivation of the *SLC5A6 *gene, which encodes the sodium-dependent multivitamin transporter SLC5A6. The aim was to develop a method for assessing the efficiency of biotin penetration across the cell membrane following inactivation of the *SLC5A6 *gene and to explore the feasibility of delivering biotin derivatives into cells independent of SLC5A6. The test system is built upon modified HEK293 cell lines with overexpression of the BirA* biotin ligase, with the first line comprising a functional *SLC5A6 *gene and the second one involving an inactivated version of this gene mimicking impaired biotin transport. This test system was used to investigate the transport of biotin and its two derivatives, namely the biotin conjugate with *p*-aminophenylalanine (Bio-1) and biotin methyl ester (Bio-2), through the cell membrane. It has been determined that biotin and its methyl ester (Bio-2) can enter cells independently of the SLC5A6 transporter, which points to the presence of alternative transport pathways. The biotin derivative Bio-1, which contains *p*-aminophenylalanine, is internalized into cells solely through the hSMVT transporter. The novel test system will serve as a tool for investigating the pathways involved in vitamin entry into cells and for developing therapeutic strategies for individuals with mutations in the *SLC5A6 *gene, as well as other transport-related genes.

## INTRODUCTION

The *SLC5A6 *gene, located at locus 2p23.3 of human chromosome 2, encodes a membrane-bound sodium- dependent multivitamin transporter (SMVT). The human hSMVT protein is composed of 635 amino acid residues and is essential for the transport of water-soluble compounds such as biotin, pantothenic acid, and alpha-lipoic acid [1]. The SMVT protein demonstrates significant evolutionary conservation and is prevalent throughout the organism. This pro

*In vivo *investigations in mice indicate that the inactivation of the *Slc5a6 *gene in intestinal cells results in growth retardation, decreased bone density, and reduced bone length, along with changes in the small intestine (villi shortening, dysplasia) and cecum (chronic inflammation, dysplasia) [6]. Therapy involving elevated dosages of biotin and pantothenic acid forestalls growth retardation and intestinal inflammation [11].

Biallelic mutations in the *SLC5A6 *gene have been observed in children with growth and developmental delays, seizures, gastrointestinal, skin, and peripheral nervous system disorders, and immunodeficiency resulting from impaired T- and B-cell function [12, 13, 14, 15, 16, 17, 18]. Clinical improvements were noted in these children, who were predisposed to infant death, after they had undergone targeted treatment with vitamins to *SLC5A6 *gene mutation carriers [13, 14, 15, 18].

For example, whole exome sequencing of a 15-month-old boy with developmental delay, microcephaly, severe immunodeficiency, and severe gastroesophageal reflux disease revealed a mutation in the *SLC5A6 *gene. At 19 months of age, the child received vitamin therapy involving high doses of biotin (10 mg/day, then 30 mg/day), pantothenic acid (250 mg/day, then 500 mg/day), and lipoic acid (150 mg/day, then 300 mg/day), with the vitamin dosages subsequently increased at 24 months. Following 14 months of therapy, the immunoglobulin levels were normalized and no bone system abnormalities remained. Comparable clinical improvement was observed in other pediatric patients who were administered high doses of biotin [19, 20].

According to our analysis of published data, studies on vitamins have not assessed the effectiveness of their absorption, distribution, and metabolism. Only a few methods are currently available for the assessment of vitamin permeation efficiency across the membrane. Typically, tritium or carbon-14 isotopes are used to label biotin for this application [21]. This methodology offers enhanced sensitivity in detecting and quantifying biotin distribution, although it requires specialized equipment for handling radioactive substances. Furthermore, this method does not facilitate the evaluation of membrane permeation of biotin derivatives, which usually do not possess a radioactive label. Biotin quantification can also be achieved using mass spectrometric analysis, which, nonetheless, requires the use of advanced analytical instruments and time-intensive procedures.

This study aimed to create a method for assessing the effectiveness of biotin permeation through the cell membrane following inactivation of the *SLC5A6 *gene. Further, we examined the possibility of delivering biotin derivatives into cells independently of SLC5A6, which could provide new avenues for patient treatment in cases of *SLC5A6 *gene mutations.

We have developed a test system to assess the efficacy of biotin penetration through the cell membrane following inactivation of the *SLC5A6 *gene. The system relies on the inhibition of biotin-carrying cellular proteins through the utilization of streptavidin and a horseradish peroxidase conjugate. Biotinylation is artificially enhanced through the application of a mutant BirA biotin ligase with reduced specificity.

The test system involves modified HEK293 cell lines that overexpress the BirA* biotin ligase. One of the lines contains a functional *SLC5A6 *gene, while in the other line this gene is inactivated. The *SLC5A6* gene is inactivated to simulate a state where biotin transport via hSMVT is impeded. The ectopic expression of biotin ligase results in the nonspecific biotinylation of proteins within the cell, which can be identified using Western blotting. Assessment of protein biotinylation levels in the cell lines following incubation with biotin or its derivatives facilitates the detection of biotin transport across the cell membrane.

The developed system was used to study the transport mechanism across the cell membrane of biotin and its two derivatives: biotin conjugate with *p*-aminophenylalanine (Bio-1) and biotin methyl ester (Bio-2).

## EXPERIMENTAL PART

**Oligonucleotide synthesis**

All oligonucleotides (primers) were synthesized by Lumiprobe RUS LLC (Russia).

**Cell cultivation**

Wild-type (WT), as well as modified (BirA*, Δ*SLC5A6,* and BirA*_Δ*SLC5A6*) HEK293, cells were cultured in a DMEM/F12 medium (Gibco, USA) supplemented with 10% (v/v) fetal calf serum (FBS HI, Gibco), 1% (v/v) L-alanine-L-glutamine (2 mM, GlutaMAX, Gibco), a 1% (v/v) antibiotic mixture (100 units/mL penicillin and 100 μg/mL streptomycin, Gibco) at 37°C and 5% CO_2_. The cells were cultivated in culture vials designed for adherent cells (25 cm²). Once the cells reached 90-100% confluency, they were split at a 1 : 10 ratio, rinsed with PBS, then detached using a trypsin-EDTA solution (1×, Gibco) in PBS, and, finally, resuspended in a fresh medium to achieve the required cell density. For the experiments, the cells were cultured in 24-well plates.

Introduction of the BirA* gene

Cells with increased biotinylated protein levels were obtained by introducing the mutant *E. coli *BirAR118G biotin ligase (BirA*) into the cells. The cell selection process involved the introduction of the BirA* gene, along with the *eGFP *gene, which encodes a green fluorescent protein from jellyfish, optimized for mammalian cells. The BirA* and *eGFP *genes were inserted using the plasmid pSBbiGN_BirA*, which was constructed previously [22] based on the pSBbiGN vector (Addgene #60517) [23].

Wild-type (WT) HEK293 cells were transfected with plasmid pSBbiGN_BirA* and plasmid pCMV(CAT)T7-SBX100 [24] that encodes a transposase, using Lipofectamine 3000, following the manufacturer’s guidelines. At 24 hours post-incubation, cells producing BirA* and eGFP were selected using a FACSAria III BD sorter and the signal was recorded at 488/530 nm. The selected cells were seeded into 96- well plates (200 µl of medium per well), followed by culturing of individual clones in 24-well plates. The resulting monoclonal cells exhibited a stable BirA* and GFP expression.

**Inactivation of the SLC5A6 gene**

The *SLC5A6 *gene in WT and BirA* cell lines was inactivated using the CRISPR-Cas9 system. The sgRNA sequences were selected for cleavage using the Benchling CRISPR design tool (https://benchling.com). The selection was made of a guide RNA targeting exon 8 of the *SLC5A6 *gene (5′-GCGGTACCTCAGTCAGTTCCCGCA-3′).

The pX459-SLC5A6 construct, designed for inactivation, was derived from the pSpCas9(BB)-2A-Puro plasmid (pX459 V2.0, Addgene #62988 [25]), which includes CRISPR/Cas9 system elements and a puromycin resistance gene. The plasmid was pre-cleaved with BpiI endonuclease to generate sticky ends.

The guide RNA-encoding sequence was synt h e s i z e d f r om t wo D N A o l i g o n u c l e o t i d e s ( 5 ′ - C AC C AC C G C G G C G G TAC C T C AG T T C C CGCA- 3′ and 5′-AAACTGCGGCGGGAACTGAGGAGGTACCGC- 3′) designed to generate complementary sticky ends (4 nucleotides) after hybridization, which would then be compatible with the sticky ends on the pX459 vector. Oligonucleotides were hybridized within a T4-DNA ligase buffer (Thermo Scientific, USA), with each added to a concentration of 1 μM, and then incubated at 95°C for 5 minutes, followed by gradual cooling to 30°C in a closed thermostat. The resulting duplex (1 μl) was ligated at sticky ends into the pX459 vector using the Rapid DNA Ligation Kit (Thermo Fisher, USA).

Following transfection of competent *E. coli* JM109 cells with the ligase mixture, the colonies were cultivated on ampicillin-supplemented plates (50 μg/mL). Plasmid DNA was purified from overnight cultures, using the Plasmid Miniprep kit (Eurogen, Russia). Sanger sequencing, with a primer positioned on the U6 promoter (5′-GACTATCATCATATGCTTACCGT-3′), confirmed the correct insertion.

In order to generate cell lines with gene-specific knockouts, the cells were transfected with plasmid pX459-SLC5A6, using the LipofectamineTM 3000 reagent (Invitrogen™: L3000001). The transfection protocol employed 100,000 cells, 1 μg of plasmid, and 1.5 μl of lipofectamine. After a 24-h incubation, the culture medium was replaced with a fresh medium including puromycin (1 μg/mL). In parallel, wild-type HEK293 control cells were incubated in a medium containing puromycin, and after 48 h, cell death was observed in all the control cells. Cells transfected with the pX459-SLC5A6 plasmid were seeded into 96-well plates (200 μL medium per well), followed by individual clone culture in 24-well plates.

**Fig. 1:**
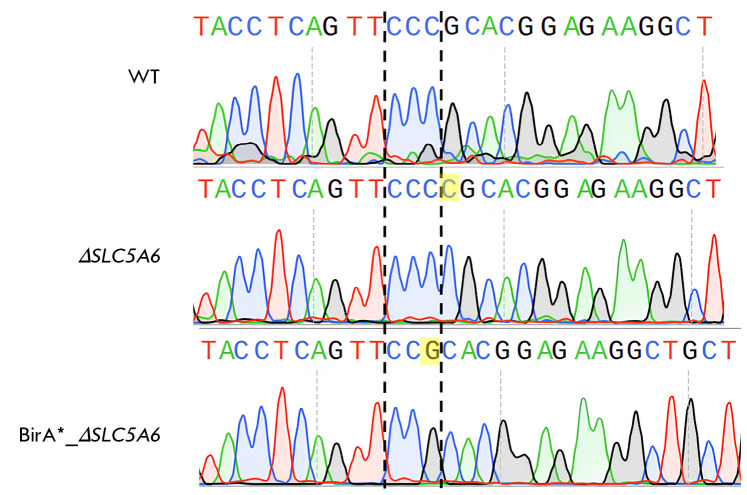
Inactivation of the* SLC5A6 *gene in HEK293 cells. Sanger sequencing results of the PCR-amplified target locus in the* SLC5A6 *gene are shown for wild-type (WT) cells, knockout cells (Δ*SLC5A6*, 1 bp insertion), and cells with the BirA* construct insertion (BirA*_Δ*SLC5A6*, 1 bp deletion)

Monoclonal lines were genotyped using total DNA extracted from the cells (QuickExtract DNA Extraction Solution, Lucigen). Subsequently, the region within the predicted cleavage site was amplified via PCR (PCR primers: 5′-CTTCTGGACCTTGGACCTTGGCCTTCGG- 3′ and 5′-GACCTTGCTCCACTCCACTCCCTTC- 3′). Sanger sequencing of amplified fragments confirmed the presence of a mutation that resulted in inactivation of the *SLC5A6* gene (*Fig. 1*). Consequently, cell lines with disrupted* SLC5A6 *reading frames were chosen for additional investigation, with a 1 bp insertion identified in the Δ*SLC5A6 *line and a 1 bp deletion identified in the BirA*_Δ*SLC5A6 *line.

**Synthesis of Bio-1**

Biotin (1.74 g, 7.13 mmol), HATU (2.71 g, 7.13 mmol), and DIPEA (2.49 mL, 14.27 mmol) were dissolved in 15 mL of anhydrous DMF via sonication. In a separate flask, a solution of 4-aminophenylalanine (2 g, 7.13 mmol) in 5 mL DMF was prepared. The biotin solution was introduced into the amino acid solution using a syringe pump with strong stirring for over an hour. Then the DMF was removed under vacuum. Under stirring, 100 mL of water was added to the residue, which was then left for one hour to precipitate. The precipitate was filtered, rinsed with H_2_O (2 × 100 mL), and then air-dried. Thus, Product 2, gray in color (3.1 g, 86%), was obtained.

**Scheme 1:**
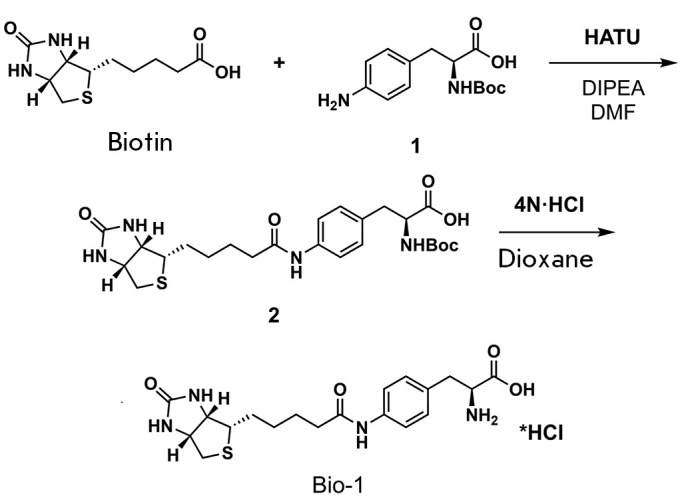
Synthesis of the **Bio-1 **compound

**^1^H-NMR **(600 MHz, DMSO-*d*6) δ = 9.8 (s, 1H), 7.5 (d, *J *= 8.0, 2H), 7.1 (d, *J *= 8.0, 2H), 7.0 (d, *J *= 8.3, 1H), 6.4 (s, 1H), 6.4 (s, 1H), 4.3 (t, *J *= 6.8, 1H), 4.3–4.1 (m, 1H), 4.1–4.0 (m, 1H), 3.2–3.1 (m, 1H), 3.0–2.9 (m, 1H), 2.9–2.7 (m, 2H), 2.6 (d, *J *= 12.4, 1H), 2.3 (t, *J *= 7.1, 2H), 1.7–1.5 (m, 3H), 1.5–1.5 (m, 1H), 1.4–1.3 (m, 1H), 1.3 (s, 9H), 1.3–1.2 (m, 1H). **^13^C-NMR **(151 MHz, DMSO-*d*_6_) δ = 173.8, 173.6, 171.0, 162.7, 155.4, 137.7, 132.5, 129.3, 118.9, 78.0, 61.1, 59.2, 55.4, 55.3, 36.2, 35.9, 28.2, 28.2, 28.1, 25.2.

Product 2, which was obtained in the preceding reaction (3 g, 5.9 mmol), was dissolved in 4 M HCl/dioxane (60 mL). The stirring of the reaction mixture for 5 h yielded a suspension. Following filtration, the precipitate was washed with Et_2_O (2 × 50 mL) and airdried, producing colorless **Bio-1 **hydrochloride (2.6 g, 98%).

**^1^H-NMR **(600 MHz, D2O) δ = 7.4 (d, *J*=8.1, 2H), 7.3 (d, *J *= 8.1, 2H), 4.6–4.5 (m, 1H), 4.4 (dd, *J *= 8.0, 4.5, 1H), 4.3 (t, *J *= 6.7, 1H), 3.4–3.3 (m, 2H), 3.2 (dd,* J *= 14.8, 7.7, 1H), 3.0 (dd, *J *= 13.0, 4.8, 1H), 2.7 (d,* J *= 13.0, 1H), 2.4 (t, *J *= 7.3, 2H), 1.7 (tt, *J *= 14.8, 7.1, 3H), 1.6–1.5 (m, 1H), 1.5–1.4 (m, 2H). **^13^C-NMR** (151 MHz, D_2_O) δ = 176.4, 171.9, 165.9, 137.1, 131.6, 130.8, 123.2, 62.7, 60.9, 56.0, 54.6, 40.3, 36.8, 35.7, 28.5, 28.3, 25.7.

**Synthesis of Bio-2**

Biotin (1 g, 4.1 mmol) was dissolved in 20 mL of methanol, then cooled to 0°C, and thionyl chloride (2 mL, 20 mmol) was subsequently added dropwise. The reaction mixture was stirred at 20°C for 10 h, and the solvent was removed in vacuo. The residue was neutralized using 1 M NaHCO_3_. The precipitate was filtered off, washed with water, and dried in air, yielding Bio-2 (939 mg, 91%) after recrystallization from acetone.

The Bio-2 spectral data were consistent with those described previously [26].

**^1^H-NMR **(600 MHz, DMSO-*d*6) δ = 6.4 (s, 1H), 6.4 (s, 1H), 4.4–4.3 (m, 1H), 4.2–4.1 (m, 1H), 3.6 (s, 3H), 3.2–3.0 (m, 1H), 2.8 (dd, *J *= 12.4, 5.1, 1H), 2.6 (d, *J *= 12.4, 1H), 2.3 (t, *J *= 7.5, 2H), 1.7–1.4 (m, 4H), 1.4–1.2 (m, 2H).**^13^C-NMR **(151 MHz, DMSO-*d*_6_) δ = 173.3, 162.7, 61.0, 59.2, 55.3, 51.2, 39.8, 33.1, 28.1, 28.0, 24.5.

**Western blotting**

Protein biotinylation efficiency was assessed at varying biotin concentrations using HEK293 WT, BirA*, Δ*SLC5A6*, and BirA*_Δ*SLC5A6 *cell lines to determine the optimal concentration. Cells from each cell line were seeded into a 24-well plate and then incubated for 24 h. Subsequently, either an aqueous solution of biotin at the appropriate concentration or a control solution (water) was added to the culture medium. The cells were further incubated with biotin for 24 h. Afterward, the cells were lysed on ice using RIPA buffer containing benzonase (Sigma, USA) for 15 minutes and the enzyme was inactivated by heating at 80°C for 3 minutes.

Western blotting was employed to analyze diluted lysates, with normalization for total protein content. Electrophoretic separation of proteins was performed in a 10% polyacrylamide gel with 0.1% SDS, followed by transfer to a nitrocellulose membrane using wet transfer (1 h at 400 mA). The membrane was blocked using a 5% skim milk powder solution [27] in TBST (1–12 h), followed by incubation for 1 h at room temperature with a streptavidin-peroxidase conjugate solution (1 : 3000 in TBST, “IMTEK”, P-S Avs, Russia). Following sequential washes with TBST (3 × 5 min), TBS (3 × 5 min), and distilled water, detection was performed using the Clarity™Western ECL substrate (Bio-Rad).

## RESULTS AND DISCUSSION

The impact of the functional activity of the multivitamin transporter SLC5A6 on biotin internalization was evaluated using the human embryonic kidney cell line HEK293. The *SLC5A6 *gene was inactivated in this cell line using the CRISPR/Cas9 system, resulting in the generation of the Δ*SLC5A6 *cell line.

**Maintenance of biotinylated biotin-dependent carboxylases in the HEK293 cell line does not require the SLC5A6 transporter**

**Fig. 2:**
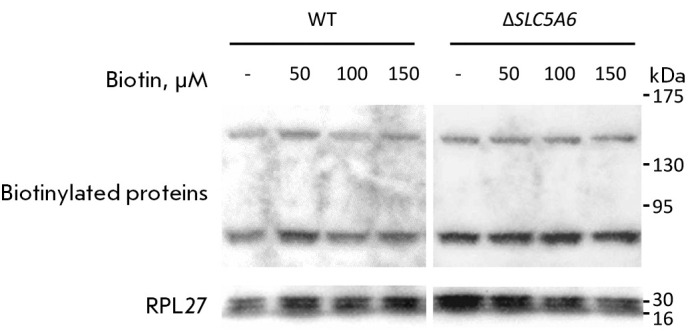
Western blotting results for HEK293 WT (left) and Δ*SLC5A6 *(right) cell lines incubated with different concentrations of biotin (50, 100, and 150 μM)

The efficiency of biotin transport across the cell membrane was assessed by comparing the levels of biotinylated proteins in the HEK293 WT and Δ*SLC5A6* cell lines. To this end, cells were incubated with biotin at different concentrations, after which biotinylated proteins were visualized by Western blotting using the streptavidin-peroxidase conjugate (Strep-HRP, *Fig. 2*). No change in the level of biotinylation was observed following the inactivation of the *SLC5A6* gene. We hypothesize that this may be due to transmembrane diffusion or endocytosis of biotin during the 24-h incubation, resulting in its comparatively elevated intracellular concentration. Moreover, other transporters, such as monocarboxylate transporter 1 (MCT1), could be involved in delivering biotin across the cell membrane [28, 29, 30]. It should be noted that Subramanian V.S. et al. formulated a hypothesis on vitamin diffusion through the membrane, which provides a rationale for the effectiveness of biotin and pantothenic acid therapy in patients with deficient multivitamin transporters [15].

**Test system for monitoring biotin permeation through the cell membrane**

Having determined that the functioning of the multivitamin transporter SLC5A6 was not a factor limiting biotin entry into cells in culture at the natural biotinylated protein content, we decided to create cell lines with artificially increased biotinylation levels.

**Fig. 3:**
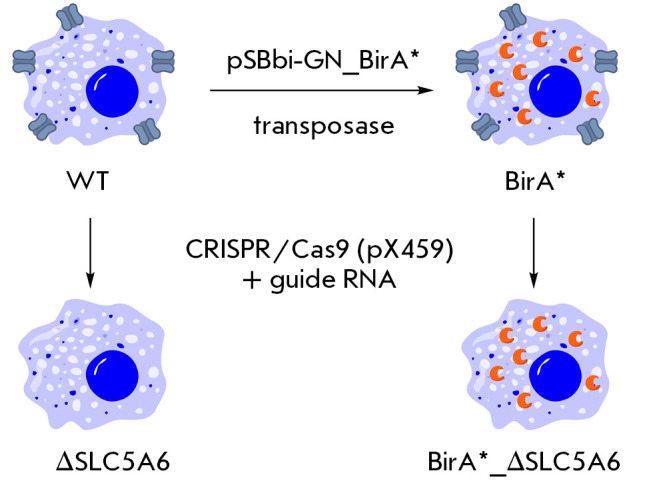
Generation of HEK293-derived cell lines. Cells with increased levels of biotinylated proteins (BirA* line) were generated by introducing the mutant biotin ligase BirA*. The BirA* gene was integrated into the genome using the pSBbi-GN_BirA* plasmid with the aid of a transposase. Inactivation of the *SLC5A6 *gene in WT and BirA* cell lines was performed using the CRISPR-Cas9 system with the pX459 vector carrying a guide RNA targeting exon 8 of the gene. As a result, Δ*SLC5A6 *and BirA*_Δ*SLC5A6 *lines were obtained

To this end, two additional cell lines were generated from HEK293 cells (*Fig. 3*).

The BirA* gene, encoding a mutant *E. coli *BirAR118G biotin ligase, was introduced into HEK293 cells using a Sleeping beauty transposase-based vector (SB100X) [31, 32]. This enzyme mediates the indiscriminate binding of biotin to lysine residues found in the protein. Consequently, the biotin that enters the cell is quickly used to biotinylate proteins that do not typically bind biotin. The level of biotinylated proteins in the cell enables one to estimate the rate of biotin penetration through the membrane.

Next, we introduced an inactivating mutation into the *SLC5A6 *gene, which encodes the hSMVT protein. This enabled us to compare the biotinylation process in cells with active and inactive hSMVT transporters. The BirA*_Δ*SLC5A6 *line was created by introducing an inactivating mutation into cells containing the BirA* gene using CRISPR/Cas9 technology (*Fig. 3*), which was similar to how the Δ*SLC5A6 *line was generated from wild-type cells.

**Assessment of biotin transport efficiency across the cell membrane using the test system**

**Fig. 4:**
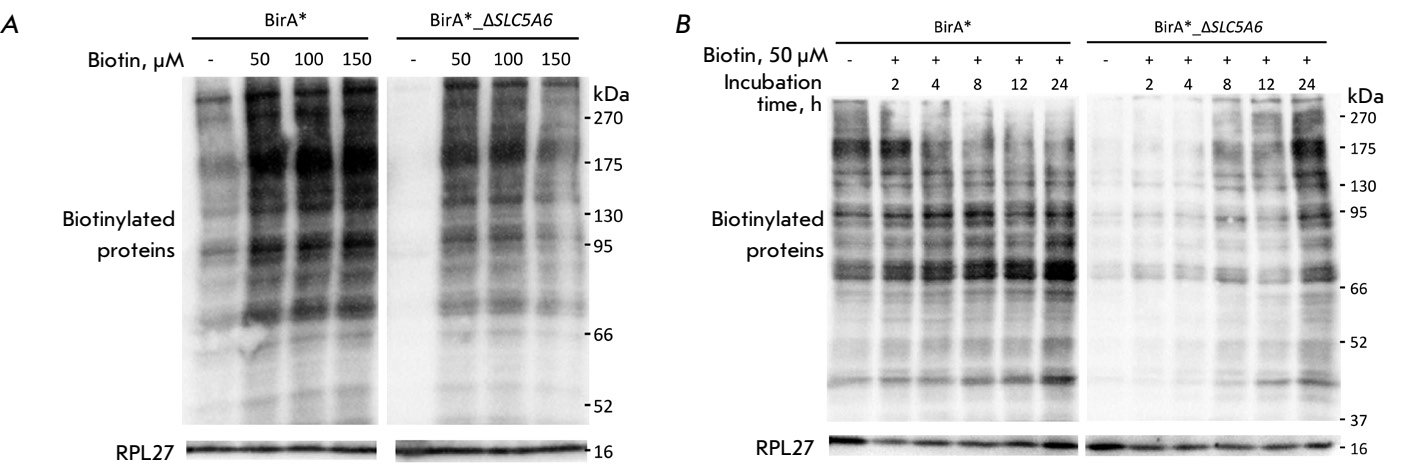
Assessment of protein biotinylation levels in BirA* and BirA*_ΔSLC5A6 cells. (*A*) Dependence of the protein biotinylation level on biotin concentration in the medium after 24-h incubation. (*B*) Dependence of the protein biotinylation level on incubation time

After establishing lines with ectopic expression of the nonspecific biotin ligase BirA*, we decided to determine the optimal biotin concentration in the medium suitable for detecting the transport of this vitamin. To this end, we incubated BirA* and BirA*_Δ*SLC5A6* cell lines with different concentrations of biotin: 0, 50, 100, and 150 μM (*Fig. 4A*) for 24 h. Both lines exhibited a significant difference in biotinylation levels when biotin was absent and at a concentration of 50 μM, followed by saturation and a further increase in biotin concentration, which did not increase biotinylation levels. Consequently, a concentration of 50 μM is the optimal concentration for the evaluation of biotin transport. Furthermore, even in the absence of specifically added biotin, the level of biotinylation was lower in cells with inactivated hSMVT than in cells with the active transporter.

Extended incubation with biotin correlated with augmented biotinylation (*Fig. 4B*), and notable disparities were evident relative to the presence of the *SLC5A6 *gene. In the first few hours of incubation, the maximum level of biotinylation in the BirA* cell line was already achieved. Concurrently, in BirA*_Δ*SLC5A6 *cells exhibiting compromised biotin transport, the accumulation of biotinylated proteins was decelerated, achieving a comparable level to the maximum observed in BirA* cells after a 24-h delay. These data suggest that hSMVT plays a critical role in biotin transport, potentially influencing the development of pathological conditions in patients with mutations in this gene.

**Synthesis of biotin derivatives for cell penetration**

The rationale for synthesizing biotin derivatives involved modifying their molecular properties to enable cell entry via alternative pathways that bypass the hSMVT transporter, which could broaden therapeutic options for individuals with *SLC5A6 *gene mutations. Two approaches were taken into consideration to accomplish this task.

**Fig. 5:**
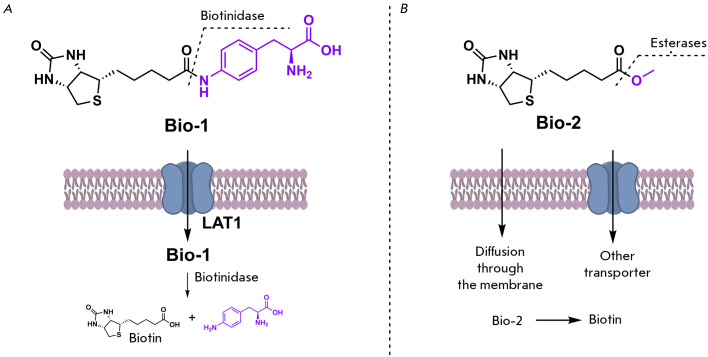
Synthesized biotin analogs Bio-1 (*A*) and Bio-2 (*B*), with the proposed mechanism of membrane transport and subsequent enzymatic cleavage leading to the release of free biotin

The first approach to delivering the molecule bypassing SLC5A6 is to create hybrid molecules (prodrugs) comprising a therapeutic part and a component that mimics a useful metabolite capable of being recognized by a specific transporter. For example, the LAT1 (Large Amino Acid Transporter-1) transporter has been successfully used to deliver ketoprofen and ferulic acid to neurons, as well as some drugs to tumor cells [33, 34, 35]. This process involves the modification of the therapeutic molecules by conjugating them to amino acids, which are LAT1 substrates. To evaluate the performance of this approach, we synthesized a biotin derivative of *p*-aminophenylalanine (**Bio-1**, *Fig. 5A*, *Scheme 1*). Our hypothesis was that upon intracellular delivery of this substance, the biotinidase enzyme would promote the release of biotin in its free form (*Fig. 5A*), as observed when biotinidase cleaves N-biotinyl-4-aminobenzoic acid into biotin and p-aminobenzoic acid [36, 37].

**Scheme 2:**
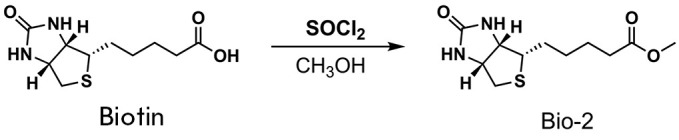
Synthesis of the **Bio-2 **compound

The second approach is to reduce the polarity of the molecule. This could either enable free diffusion of the molecule across the membrane or activate a different transporter, which would render hSMVT unnecessary. We synthesized a biotin methyl ester (**Bio-2**) that exhibits enhanced hydrophobicity. After entering the cell, biotin can be released by the action of esterases (*Fig. 5B*, *Scheme 2*).

Cellular permeability of biotin and its derivatives was assessed by incubating biotin, Bio-1, or Bio-2 with HEK293 (WT), Δ*SLC5A6*, BirA*, and BirA*_Δ*SLC5A6* cell lines. All three molecules were shown to serve as a source of biotin in cells with a functional hSMVT transporter. Hence, no disparities in protein biotinylation levels were apparent in wild-type cells exposed to biotin, Bio-1, and Bio-2 (data not shown).

**Fig. 6:**
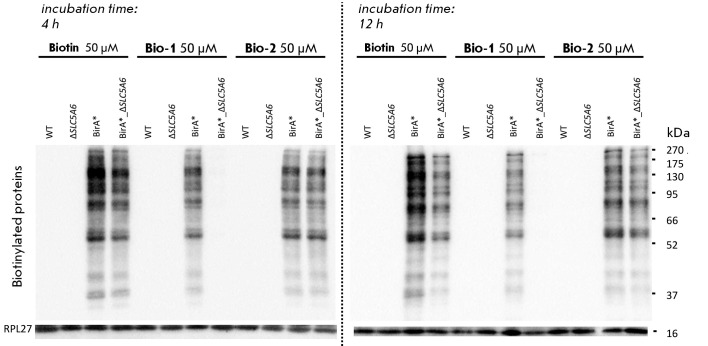
Comparison of protein biotinylation levels in various cell lines after incubation with biotin, Bio-1, and Bio-2

When cells with ectopic expression of BirA* biotin ligase were used, the level of protein biotinylation increased manifold. Under these conditions, each of the three molecules can be used to transport biotin into the cell (*Fig. 6*, BirA* line). A modest reduction in membrane permeation rates was observed for Bio-1 and Bio-2 compared to biotin. In all cases, saturation was observed after 4 h of incubation.

Upon hSMVT inactivation, biotin entry into the cell was reduced. After both 4 h and 12 h of incubation, the level of biotinylated proteins in BirA*_Δ*SLC5A6* cells was observed to be lower than in BirA* cells.

The cell incubation with Bio-1 yielded surprising outcomes: inactivating hSMVT prevented the increase of biotinylated proteins in cells even after 12 h of Bio-1 exposure (*Figure 6*, BirA*_Δ*SLC5A6 *cells), but biotinylation remained high when a functional transporter was present (*Figure 6*, BirA* line). Based on these findings, it is reasonable to conclude that the transport mechanism of Bio-1 does not involve LAT1, contrary to the initial hypothesis. The molecule in question probably can enter the cell solely through hSMVT participation, accounting for the high biotinylation level in BirA* cells and the absence thereof when the SLC5A6 gene is inactivated. Therefore, the effect we observed was contrary to our expectations. It was found that biotin can enter cells through several pathways, with the pathway via hSMVT being only one of many. In contrast, the Bio-1 derivative proved unable to use the transport pathway available to biotin and could enter cells only via hSMVT.

Conversely, when cells were incubated with Bio-2, the disparity in protein biotinylation between BirA*_Δ*SLC5A6 *cells and BirA* cells was negligible. These data indicate that the cellular penetration of the Bio-2 compound does not rely on the hSMVT transporter.

These findings suggest that biotin within the Bio-1 molecule is crucial for transporting related fragments via hSMVT. At the same time, *p*-aminophenylalanine coupled with biotin does not affect transport via hSMVT, but it does interfere with other transport pathways. This property finds application in targeted drug delivery into cells [38, 39] in the form of a biotin conjugate. The hSMVT protein is believed to be essential for the transport of these drugs. However, despite extensive research, several questions regarding the mechanism of transport of these conjugates remain unanswered [30]. For instance, research [40] has shown that an unbound carboxyl group in the biotin compound is necessary for its effective movement through the SMVT. Nevertheless, in studies positing SMVT-mediated prodrug transport, biotin was attached to the conjugate only through the carboxyl group [30]. Our findings also indicate that the free carboxyl group of biotin is not required for the transport of biotin derivatives via hSMVT.

The newly developed test system enabled us to demonstrate that biotin and its methyl ester Bio-2 could be transported into cells without the involvement of hSMVT. We anticipate that our test system will be instrumental in developing biotin-containing prodrugs.

## CONCLUSION

This work introduces a new system for monitoring the cellular transport of biotin and its derivatives. This system offers an alternative to intricate methodologies involving radioactively labeled biotin.

Using this novel test system, we determined that biotin and its methyl ester (Bio-2) can permeate cells independently of the hSMVT transporter, encoded by the *SLC5A6 *gene, implying the existence of other methods of transportation. However, as cellular biotin demands increase, hSMVT becomes critical for efficient delivery.

The cellular uptake of the biotin conjugate with* p*-aminophenylalanine (Bio-1) is mediated solely by hSMVT, rendering it incompatible with alternative delivery pathways. Nevertheless, this specificity enables hSMVT to be used to transport other compounds into cells when conjugated with biotin. The developed test system is an important tool for investigating the process of vitamin uptake by cells, potentially enabling the development of treatment strategies and the assessment of drug efficacy in patients with *SLC5A6 *gene mutations and other transporter deficiencies.

## Abbreviations

Strep-HRP: streptavidin conjugated with horseradish peroxidase
SLC5A6 or SMVT: sodium- dependent multivitamin transporter
BirA*: mutant biotin ligase from E. coli (BirA R118G)
PBS: phosphate-buffered saline
GFP: green fluorescent protein
